# Content Analysis of Human Anatomy Mobile Apps for Undergraduate Student Learning using MARuL

**DOI:** 10.1007/s40670-026-02649-z

**Published:** 2026-02-20

**Authors:** Husna Zahirah Md Reshad, Syahrir Zaini, Nurul Asyiqin Yusof

**Affiliations:** 1 Kulliyyah of Pharmacy, International Islamic University Malaysia, Jalan Sultan Ahmad Shah, 25200 Kuantan, Pahang Malaysia; 2 Department of Pharmacy Practice, Kulliyyah of Pharmacy & Qualitative Research Group, Kulliyyah of Pharmacy, International Islamic University Malaysia, 25200 Kuantan, Pahang Malaysia; 3 Department of Basic Medical Sciences (Anatomy Division), Kulliyyah of Pharmacy, International Islamic University Malaysia, Jalan Sultan Ahmad Shah, 25200 Kuantan, Pahang Malaysia

**Keywords:** Mobile apps, Anatomy, MARuL, App quality, Student learning

## Abstract

**Introduction:**

Mobile applications (apps) have gained prominence in health education, particularly for teaching human anatomy, by offering interactive and personalised platforms that enhance learning for both educators and students. Mobile Application Rubric for Learning (MARuL), a validated assessment tool, was designed to rate the quality of educational apps focusing on the teaching and learning aspects. This study aimed to systematically search and evaluate the quality of human anatomy apps for student learning using MARuL.

**Methods:**

The iOS App Store was searched using related keywords. The inclusion criteria were: English language, focus on human anatomy learning, non-gaming apps, a star rating of 4.0 or above, and availability of a free version. Two trained reviewers rated the apps independently. Results were computed using Jamovi 2.3.28 and Microsoft Excel (version 16).

**Results:**

The systematic search resulted in 20 relevant apps for evaluation. The reliability measures between the two raters in the overall MARuL score showed good reliability with ICC = 0.821 (95% CI: 0.602–0.925). The top three apps with the highest MARuL score are *BioDigital Human 3D Anatomy*, *TeachMeAnatomy* and *Anatomyka Atlas*. Among the four sections assessed in MARuL evaluation (Teaching and Learning, User-centred, Professionalism, and Usability), the Usability section emerged as the highest-rated domain, largely because most apps focused on aesthetics, functionality, ease of use, and technical specifications.

**Conclusion:**

The three highest-scoring apps demonstrated high quality and fulfilled the criterion as *probably valuable* according to the MARuL scale. Future research should assess the effectiveness of top-rated apps in undergraduate anatomy pedagogy.

## Introduction

Rapid technological advancement driven by Industrial Revolution 4.0 and the Internet of Things (IoT) has transformed higher education from traditional teaching to blended, technology-based learning. According to Galvis [1], technology-enhanced learning improves educational delivery and quality, particularly for undergraduate students who are mainly from Generation Z, and soon, Generation Alpha. These generations are characterised as digital natives and are highly proficient with technology [2]. They prefer interactive, visual, and game-based learning approaches. However, effective implementation of digital learning requires adequate training and access to technology [3].

According to Xu et al. [4], 95.8% of medical students at Chongqing Medical University own a smartphone for academics, and 96.8% of them use it during lectures, classes, and meetings. This implies that almost all students utilise mobile devices for communication and as essential tools for accessing academic resources and enhancing their medical education. The widespread use of mobile devices has led to a rapid growth of mobile applications (apps) [5] for entertainment, social media and education. These apps can be installed from mobile app stores like the iOS App Store or Google Play Store. In recent decades, educational apps have become available in various subjects [6], including medical education fields such as surgery, pathology, and anatomy [5].

Human anatomy is a fundamental course in the medical and healthcare education, requiring students to memorise extensive terminology and complex structures [7]. To support learning, many undergraduates, such as medical, dental, pharmacy, nursing, and allied health sciences students, use supplementary tools beyond textbooks. Nowadays, mobile apps for human anatomy are widely used among medical students [4], with most perceiving them as useful for learning and academic performance. Eladl et al. found a high usage rate of anatomy apps among medical students at the University of Sharjah, UAE [8]. These apps offer ease of access, flexibility, and personalised learning experiences [7]. Traditional classrooms often overlook the diverse learning styles, limiting student comprehension and engagement [9]. Reliance on 2D diagrams further restricts the visualisation of complex anatomy compared to 3D approaches [10, 11].

In evaluating app quality, most research employed the well-established Mobile Application Rating Scale (MARS) [12] to assess the quality of apps in terms of engagement, functionality, aesthetics, and information quality [13–16]. The most recent study was by Garcia et al. [11], who conducted a systematic search on human anatomy mobile apps in the Google Play Store, evaluating their general quality using the MARS scale. However, the scale is less effective for evaluating teaching quality and learning elements. As compared to MARuL, MARS does not evaluate the user feedback on the app’s impact and effectiveness on learning, its alignment with learning objectives, and its relevance to course material. Nevertheless, MARS is an excellent scale for evaluating apps’ general and aesthetic quality and has been widely used in numerous health-related apps [15–18].

On the other hand, a recently developed scale, Mobile Application Rubric for Learning (MARuL), was developed to evaluate the quality of mobile apps and their effectiveness in supporting student learning [19]. The detailed criteria and scoring description for each MARuL item are outlined in Gladman et al. [19]. MARuL focuses on criteria crucial for learning, such as content accuracy, alignment with learning objectives, credibility, usability, functionality, and student engagement. It consists of four sections encompassing 26 items: (Part A) Teaching and Learning, (Part B) User-centred, (Part C) Professionalism, and (Part D) Usability [20], providing a structured framework to assess the educational value of anatomy apps for undergraduate student learning. Despite the widespread use of anatomical apps among medical students, few have been evaluated using a validated scale [21]. Therefore, this study aimed to systematically search and evaluate the quality and educational value of human anatomy apps using the MARuL scale, identifying the three highest-quality apps and the highest-scoring MARuL domain.

## Methods

**Fig. 1:**
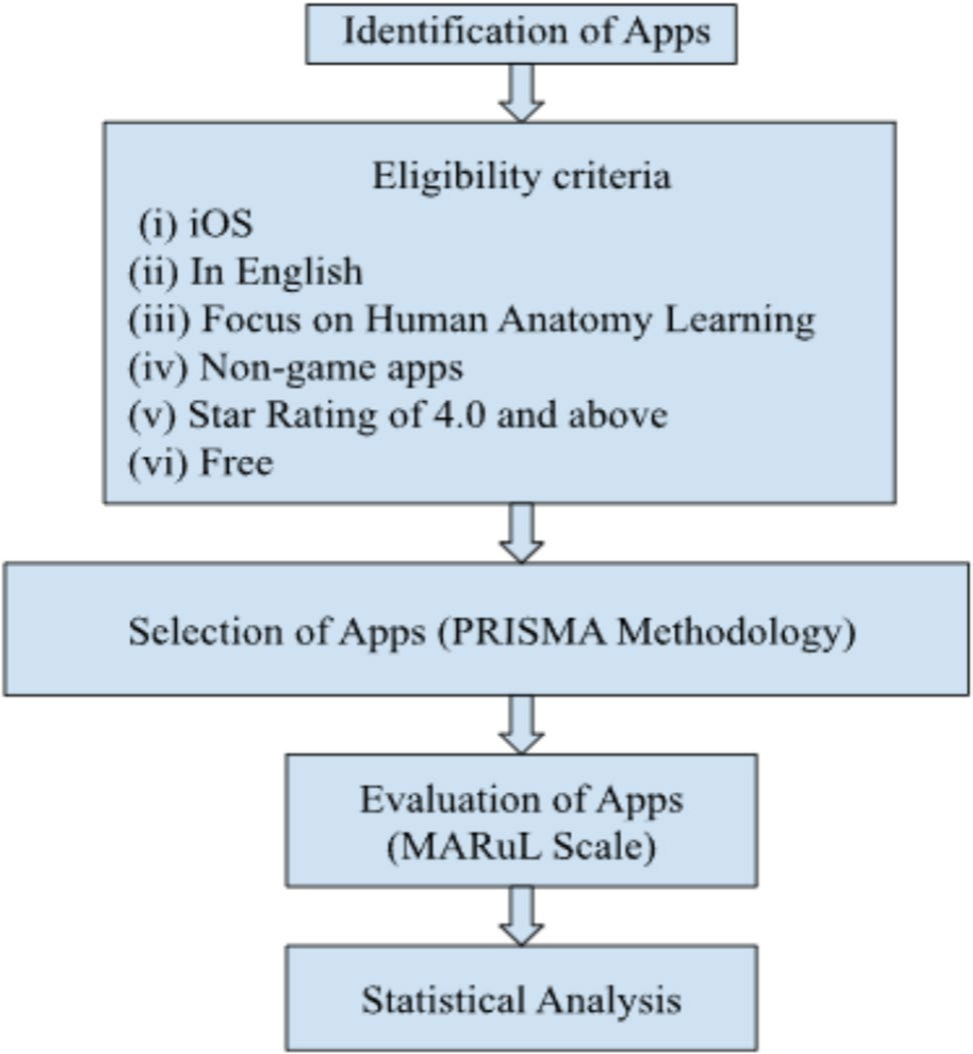
The methodological framework for this study

This study reported on the use of the MARuL scale to evaluate human anatomy apps intended for student learning. A summary of the methodology used is presented in a flowchart (Fig. 1).

### App Identification

A systematic app search was performed in the Apple App Store from 7th October to 22nd November 2024. The Apple App Store was selected to address a gap in existing research, as previous studies on human anatomy mobile apps, including Garcia et al., have focused on the Google Play Store [11]. Anatomically related keywords were used to identify mobile apps focused on learning and teaching. The search terms used were “anatomy” and “human anatomy”. During the search, apps were extensively screened by examining their names and descriptions in the app store. The iOS store was accessed using an iPhone 13 Pro Max running iOS version 17.6.1.

#### Eligibility Criteria

The inclusion criteria of the apps were as follows: (1) available on the Apple App Store, (2) in English language, (3) related and focused on learning human anatomy, (4) non-game apps, (5) star rating of 4.0 or higher, and (6) a free version.

#### App Selection

**Fig. 2:**
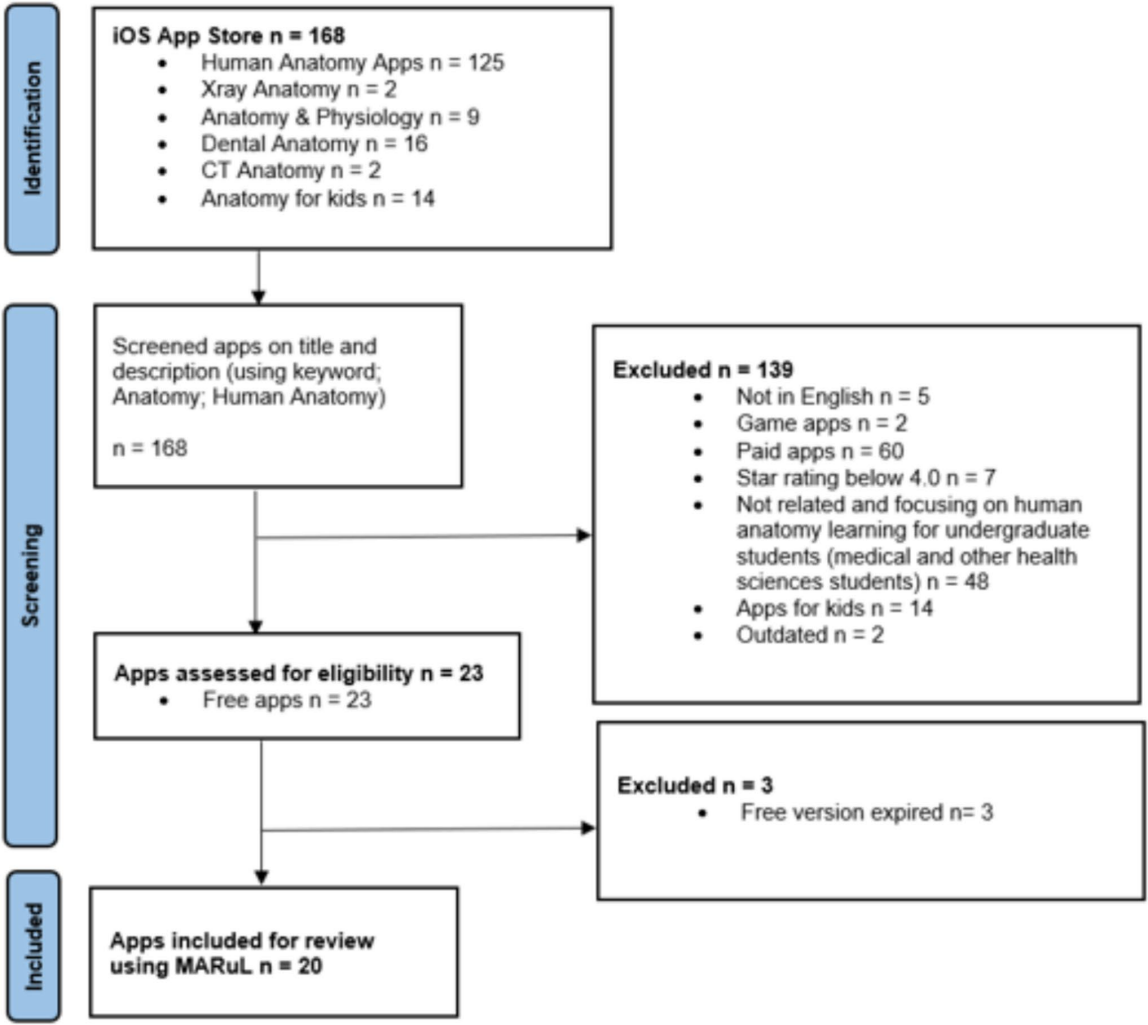
PRISMA flow diagram for identification, screening, and selection of human anatomy apps in the iOS App Store

The Preferred Reporting Items for Systematic Reviews and Meta-Analyses (PRISMA) methodology (Fig. 2) was adopted in the search and screening process for human anatomy mobile apps, as outlined in Jannati et al. [15]. PRISMA comprises three distinct phases: Identification, Screening and Inclusion.

Following the identification of mobile apps relevant to anatomy through specific keywords, these apps were subsequently screened for eligibility based on predetermined inclusion criteria. Eligible apps were then selected for review and assessed using the MARuL framework. During the search, the characteristics of the selected app, such as its name, identification screen, developer, app version, cost, app size, last update, and description, were documented in Table 1.
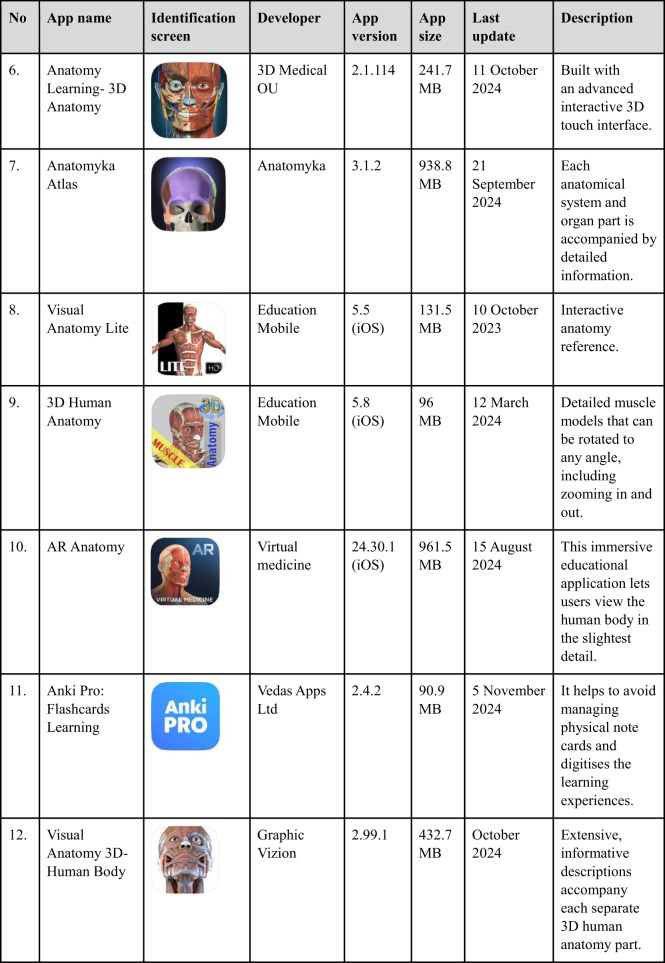

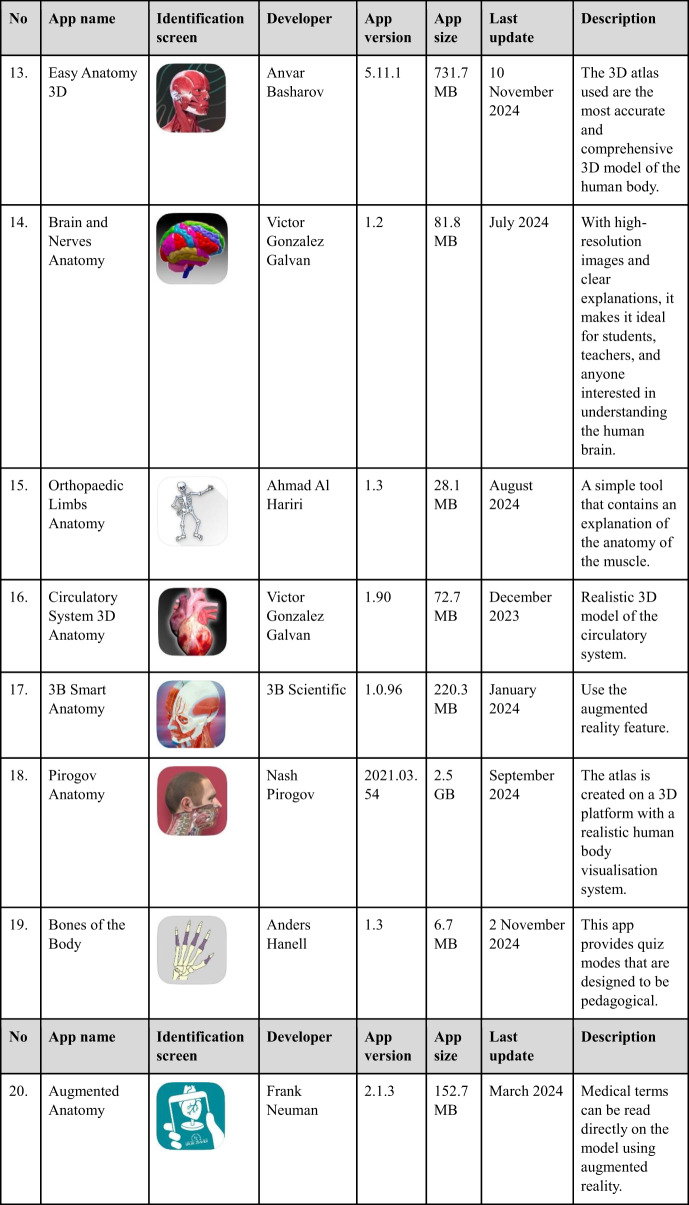

**Table 1:**
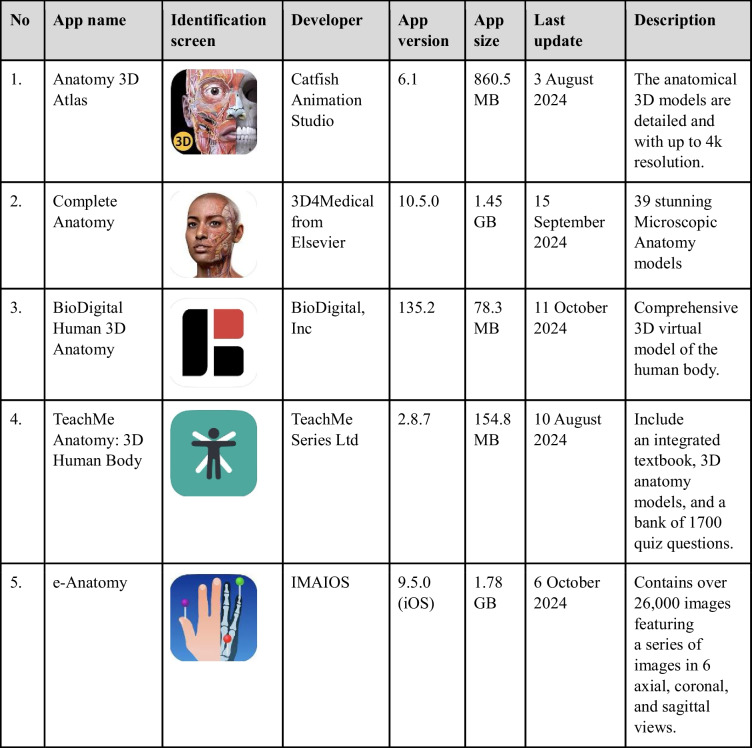
Characteristics of selected human anatomy mobile apps (Apple App Store)

#### MARuL evaluation scale

The Mobile App Rubric for Learning (MARuL) is a comprehensive evaluation tool comprising four distinct sections, each containing specific items to assess educational quality [19]. In total, the scale consists of 26 items distributed as follows:

- Part A: Teaching and Learning (9 items) Assess the app’s alignment with learning objectives, accuracy of content, depth of explanation, support for understanding, and ability to facilitate meaningful learning.
- Part B: User-centred (7 items) Evaluates aspects such as learner engagement, adaptability to user needs, and the overall learning experience.
- Part C: Professionalism (3 items) Examines the credibility of the app, including authorship, referencing, currency of information, and ethical and professional standards.
- Part D: Usability (7 items) Focuses on technical performance, ease of navigation, functionality, layout, and overall user interface design.

For this study, 20 selected apps were evaluated against these 26 items. Each item was rated on a 5-point Likert scale, ranging from 0 (does not fulfil the item requirements) to 4 (fully meets requirements). To determine the final evaluation, scores from the total of 26 items were summed to produce a total possible score of 104. Based on this, the educational value of each app was categorised using the threshold defined by Gladman et al. [20]: < 50 (Not valuable at all), 51–69 (Potentially valuable), and > 69 (Probably valuable). Additionally, the average scores for all 20 apps were calculated across the four sections to identify which domains received the highest and lowest ratings from the reviewers.

#### App Rating

All the included apps were independently rated by two reviewers (HZR and NAY) using MARuL, adapting the study by Gladman et al. [20]. One of the reviewers (HZR) is a final year undergraduate pharmacy student who has experienced learning an anatomy course, while the other independent reviewer (NAY) is an anatomist who has been teaching the subject for more than 10 years.

Firstly, before rating the apps, the two reviewers had a face-to-face meeting to confirm their understanding of each rubric and its categories. Then, they completed a pilot rating on three random anatomical apps and met up to discuss their scoring on items and how to interpret items that they differed on. The reviewers independently downloaded and reviewed the apps in iOS: iPhone 13 Pro Max (HZR) and iPad 9^th^ generation (NAY) between 25^th^ November and 27^th^ December 2024. The reviewers familiarised themselves with each of the apps for at least 10 min, with the average exploration around 15–30 min, especially for apps with many features and functions, before evaluating using MARuL. The average time was comparable to that reported in the previous study [22]. The reviewers periodically revisited the applications during the rating process to verify specific functions and ensure scoring accuracy. After the app's reviews were completed, the data were recorded and exported to an Excel spreadsheet (version 16). For each app, both section scores and overall scores on MARuL were calculated for each reviewer. To measure interrater reliability, the intraclass correlation coefficient (ICC) was computed for the overall MARuL scores and each section (Parts A, B, C, and D) between the two raters, including their 95% CI in Jamovi 2.3.28 based on a two-way random effects model [23].

## Results

### App Store Search

The search and screening of the human anatomy apps followed the Preferred Reporting Items for Systematic Reviews and Meta-Analyses (PRISMA) methodology, resulting in 20 free, English-language iOS apps meeting the inclusion criteria, after exclusion based on relevance, cost, target users, ratings and availability (Fig. 2). Free apps were selected to suit the target users of anatomy mobile apps (undergraduate students), who may have limited budgets, consistent with the approach adopted by Garcia et al. [11].

### Assessment Reliability

Table 2 presents the interrater reliability measures between the two raters for the overall MARuL score and its four sections across the 20 apps. Reliability was assessed using a two-way random-effect model with absolute agreement, as recommended by Koo & Lee [23]. Based on their guidelines, the overall MARuL score demonstrated *good reliability* (ICC = 0.821; 95% CI: 0.602–0.925). Individually, *good reliability* was observed in the Teaching and Learning section (Part A; ICC = 0.826) and the Professional section (Part C; ICC = 0.858), while the User-centred section (Part B) showed *moderate reliability* (ICC = 0.718). In contrast, the Usability section (Part D) exhibited *poor reliability* (ICC = 0.492), indicating substantial variability between raters. While all p-values were statistically significant (p < 0.05), the interpretation of reliability was primarily based on ICC values and their 95% confidence intervals. The relatively wide confidence interval across sections suggests limited precision, likely due to the small sample size and the restricted number of raters.

**Table 2:** Interrater reliability score for the overall MARuL score and the four MARuL sections' scores

MARuL score	Intraclass correlation (ICC)	95% CI	*F* test (df)	*P* value
Overall	0.821	0.602–0.925	10.2 (19.0)	< 0.001
Teaching and Learning (Part A)	0.826	0.612–0.927	10.5 (19.0)	< 0.001
User-centred (Part B)	0.718	0.413–0.878	6.08 (19.0)	< 0.001
Professional (Part C)	0.858	0.677–0.941	13.1 (19.0)	< 0.001
Usability (Part D)	0.492	0.0755–0.763	2.94 (19.0)	< 0.05

### App Rating

Table 3 presents the overall results of the 20 apps reviewed by the two raters. The average overall MARuL scores for all human anatomy apps ranged from 33.5 to 71.5 (Table 3). The top three apps, *BioDigital Human 3D Anatomy*, *TeachMeAnatomy*, and *Anatomyka Atlas* achieved the highest MARuL scores (greater than 69 out of 104) and were classified as *probably valuable*. These three apps received an identical MARuL score of 71.5.

**Table 3:** Average total MARuL score and section scores from two raters for the 20 rated apps

Score	Total score out of 104	Teaching and learning (Part A) out of 36	User-centred (Part B) out of 28	Professional (Part C) out of 12	Usability (Part D) out of 28	MARuL score category
Apps name						
BioDigital Human 3D Anatomy	71.5	23	19.5	10	19	*Probably valuable*
TeachMe Anatomy	71.5	26	21	6	18.5	
Anatomyka Atlas	71.5	24.5	18.5	8	20.5	
Anatomy 3D Atlas	59.5	17	17	6	19.5	*Potentially valuable*
Visual Anatomy 3D	59.5	20	14.5	7	18	
AR Anatomy	57.5	16.5	17	7	17	
Bones of the Body	55	19	15	2.5	18.5	
Easy Anatomy	52.5	18.5	14.5	3	16.5	
Anatomy Learning	51	17	16	2.5	15.5	
3D Human Anatomy	49	16.5	14.5	4	14	*Not valuable at all*
Complete Anatomy	48	8.5	12.5	7.5	19.5	
Pirogov Anatomy	47.5	13.5	15	2.5	16.5	
3B Smart Anatomy	45.5	18	10	2.5	15	
Orthopaedic Limbs Anatomy	44.5	12.5	9	10.5	12.5	
e-Anatomy	41	9	11	3.5	17.5	
Visual Anatomy Lite	40.5	11.5	10	4	15	
Anki Pro Anatomy	38	11.5	7	3	16.5	
Augmented Anatomy	35	9.5	6	4.5	15	
Brain and Nerves	34.5	13	7.5	4	10	
Circulatory System	33.5	12	7	3.5	11	

Subsequently, six apps: *Anatomy 3D Atlas*, *Visual Anatomy 3D*, *AR Anatomy, Bones of the Body, Easy Anatomy*, and *Anatomy Learning*, were classified as *potentially valuable*. The remaining 11 anatomy apps scored below 50 and were classified as *not valuable at all*. Among these, *Augmented Anatomy, Brain and Nerves*, and *Circulatory System* received the lowest scores of 35, 34.5, and 33.5, respectively.

Among the 20 apps, *TeachMeAnatomy* achieved the highest score in both the Teaching and Learning Sect. (26/36) and the User-centred Sect. (21/28). *Complete Anatomy* obtained the lowest score in the Teaching and Learning Sect. (8.5/36), while *Augmented Anatomy* had the lowest score in the User-centred Sect. (6/28).

In the Professionals section, *Orthopaedic Limbs Anatomy* scored the highest (10.5/12). In contrast, the lowest score (2.5/12) was recorded by *3B Smart Anatomy, Bones of the Body, Anatomy Learning*, and *Pirogov Anatomy*. Finally, in the Usability section, *Anatomyka Atlas* achieved the highest score (20.5/28), whereas *Brain and Nerves* received the lowest score (10/28).

As summarised in Table 4, a comparison of the four MARuL sections (Teaching and Learning, User-centred, Professionals, and Usability) revealed that Usability received the highest overall rating, followed by the User-centred, Teaching and Learning, and Professional sections. Each of the four MARuL sections comprises several items, with each item scored from 0 (lowest) to 4 (highest). For the 20 apps evaluated, the minimum score for an item is 0, and the maximum possible score is 80. Across the four domains, the highest-scoring items were within the Usability domain: Item 24 (Advertisement), Item 25 (Technical specifications), and Item 21 (Functionality), with average scores of 64 ± 1.4, 57 ± 2.8, and 56 ± 2.8, respectively. In contrast, Item 22 (App Differentiation) received the lowest mean score of 5.

**Table 4:** The average total MARuL score of the 20 apps and the four sections

Section	Item	Average total score (20 apps)	SD
Teaching and Learning (Part A) (35.2 ± 6.5)	Item 1: Purpose	25	1.4
	Item 2: Pedagogy	46	2.8
	Item 3: Capacity to generate learning	39	0.0
	Item 4: Quantity of information	43	4.2
	Item 5: Relevance to study/course	44.5	3.5
	Item 6: Instructional features	32.5	2.1
	Item 7: User interactivity	28	2.1
	Item 8: Feedback	16.5	2.1
	Item 9: Efficiency	37.5	3.5
User-centred (Part B) (37.5 ± 6.9)	Item 10: Subjective quality	40.5	3.5
	Item 11: Satisfaction	33	1.4
	Item 12: Perceived usefulness	39	1.4
	Item 13: Perceived importance	36.5	2.1
	Item 14: User experience	37	0.0
	Item 15: Intention to reuse	42	4.2
	Item 16: Engagement	31.5	2.1
Professional (Part C) (33.8 ± 6.7)	Item 17: In line with professional standards	50.5	0.7
	Item 18: Credibility of the app	25	0.0
	Item 19: Information quality	21.5	3.5
Usability (Part D) (46.5 ± 7.2)	Item 20: Aesthetics	46	4.2
	Item 21: Functionality	56	2.8
	Item 22: Differentiation	5	0.0
	Item 23: Ease of use	52.5	9.2
	Item 24: Advertisements	64	1.4
	Item 25: Technical specifications	57	2.8
	Item 26: Advantages of using the app over web-based or conventional equivalent	40	1.4

## Discussion

### Principal Findings

Mobile apps for teaching and learning human anatomy are widely used among medical and health sciences students [4]. In this study, a systematic search of the iOS App Store for anatomy mobile apps for undergraduate learning identified 20 relevant apps. Subsequent verification revealed that 19 out of 20 selected apps (except app no. 11: Anki Pro) are also available on the Google Play Store, making them widely accessible to students on either the Android or Apple platforms. The quality of these apps was evaluated using the MARuL scale, which was selected because it offers a more comprehensive and objective assessment compared to general app evaluation methods [24].

Two trained raters assessed app quality using the MARuL scale, with final scores based on their average ratings. This dual-rater methodology has been documented in other studies [20, 25]. The reliability measures from the two raters on the average total MARuL score and three (Part A, B and C) sections revealed a good ICC value, which could be attributed to the complementary expertise and similar health background of the raters. Nevertheless, the Usability (Part D) section yielded a low ICC score, indicating *poor reliability*. This could be due to the highly subjective nature of the items within this domain. Aspects such as aesthetics and ease of use are dependent on individual preferences, which, as noted by Zamli et al., can vary significantly even between raters from a similar discipline [26]. Consequently, this inherent subjectivity reduces the consistency of evaluations, leading to a lower level of measurement reliability for this section. Additionally, the two raters used different Apple devices (iPhone and iPad), which may have influenced user experience and usability ratings. However, the acceptable total inter-rater reliability score indicates that app quality, rather than device type, primarily influenced the ratings. Evaluating apps across different device types reflects real-world usage patterns and may enhance the generalisability of the findings.

Among the 20 apps evaluated, only three achieved high scores and were classified as ‘probably valuable’ according to MARuL scale: *BioDigital Human 3D Anatomy*, *TeachMeAnatomy*, and *Anatomyka Atlas*. Although these top three apps differ from those of Garcia et al., probably due to distinct evaluation frameworks, these apps were arguably high-performing in both studies, reinforcing their overall quality [11]. In this study, the three apps scored highly across all four sections and excelled in features that are effectively engaging students, consistently providing educational value and enhancing user experience.

For instance, the *Anatomyka Atlas* offers high-quality images of human bones and includes an interactive instructional guide to help users navigate its features. In contrast, the three apps with the lowest overall scores are *Augmented Anatomy, Brain and Nerves*, and *Circulatory System* and were classified as ‘not valuable at all’. This suggests that these apps possessed inadequate essential content or substandard image quality, making them ineffective for supporting undergraduate students' learning of human anatomy.

Table 4 presents the average total MARuL scores of the 20 apps for each item, with the average total score for each section subsequently. In the Teaching and Learning (Part A) section, *TeachMeAnatomy* obtained the highest score due to its established learning goals, which are relevant to anatomical pedagogy. Furthermore, the apps generate meaningful learning experiences, provide comprehensive information, and are robust enough to be used as a standalone study resource. A previous study demonstrates that an anatomy mobile app has a direct impact on academic success [27], which justifies the importance of this section in app evaluation. In contrast, the lowest-scoring app in this section was *Complete Anatomy*. This result may be attributed to the labelling of specific structures, for example, parts of a bone, being listed on the left side of the interface, rather than pointing directly to the anatomical structures. Additionally, the evaluation was limited to the app’s free version, which lacked interactive features such as quizzes.

For the User-centred (Part B) section, *TeachMeAnatomy* once again achieved the highest score. In addition to its excellent performance in the Teaching and Learning (Part A) section, the app is consistent with user needs effectively. The app provides an intuitive, engaging, and user-friendly experience, which suggests a high likelihood of continued student use. Conversely, the lowest-performing app was *Augmented Anatomy*. The app’s ability to optimise user experience and satisfaction is limited, primarily due to the layout design that could be more user-friendly. For instance, while the inclusion of augmented reality (AR) is a good strength, users may face difficulties due to the current layout and instructional guidance.

Subsequently, the top-rated app in the Professional (Part C) domain was *Orthopaedic Limbs Anatomy*. This app provided clear information on the involvement of qualified medical professionals and academics. In contrast, the lowest-rated app in this section was *3B Smart Anatomy,* as it did not provide information on the qualifications of the developers and limited information on the references. *Anatomyka Atlas* achieved the highest score in the Usability (Part D) section. The app features high-quality images and an intuitive layout. For example, students can enlarge specific parts of a bone and select individual structures to assess detailed information. Besides working very well, this app provides comprehensive support and responds promptly to technical issues. In contrast, *Brain and Nerves* received the lowest score, reflecting opportunities for improvement in its layout. Users may find the labelling of brain structures difficult to interpret, while the interface constrained the efficiency of navigation when exploring the app's function.

Comparing the four sections in MARuL, this study found that the Usability (Part D) section scores the highest rated domain. In this domain, several apps scored high in functionality (Item 21), which analyses the app’s speed and performance. Compared with the MARS evaluation, this result is consistent with Gladman et al., who revealed that most apps scored the highest in the functionality category [20]. Conversely, the low score for Item 22 (app differentiation) in the Usability section could reflect the widespread lack of adaptability to individual learning needs in the current apps. Future developers could focus on enhancing app differentiation by incorporating customisable difficulty levels to better suit individual learning needs.

Meanwhile, the lowest-rated domain in the MARuL scale was Professionalism (Part C), probably due to developers often prioritising user experience and commercial viability over providing evidence-based content. This finding aligns with Garcia et al. [11], who reported high functional and aesthetic scores for anatomy apps, but significant deficiencies in information quality when evaluated via the MARS scale. App developers should prioritise pedagogical rigour to bridge the current gap between high usability and functionality with educational integrity.

Finally, the authors propose reclassifying items 6,7,8,18,19, 24, and 25 into a ‘fixed-score’ category. These items are based on factual and app-specific characteristics such as instructional features, assessment tools (e.g. quiz), credibility (references), technical specification (updates), and advertisement visibility. The evaluation of these items does not depend on the rater’s personal perspective; hence, scoring for these items in MARuL remain constants across raters. Additionally, Aldekhyyel & Almulhem highlighted the absence of open-ended questions, which could have potentially addressed the evaluator’s concern and feedback more effectively [24].

### Limitations

This study has several limitations that should be acknowledged. First, some of the reviewed apps may have been updated since the MARuL evaluation, potentially altering their features and functionality and affecting future analysis. Additionally, this study excluded paid mobile apps, which may have limited the comprehensiveness of the evaluation, as they often offer more advanced features and have fewer usage restrictions. Consequently, the findings may not fully represent the entire spectrum of anatomy apps available in the market. Nevertheless, as the target users are undergraduates, free apps are more accessible to students with limited financial resources. Next, the search strategy relied on two primary keywords (‘anatomy’, ‘human anatomy’), which may not capture the available apps with other terminology, such as ‘clinical’ or ‘3D’ anatomy. Furthermore, the inclusion of only 20 free apps and a limited number of raters likely contributed to the relatively wide confidence intervals and limited precision of the interrater reliability estimates. Lastly, one item within the MARuL scale is specifically designed to evaluate clinical skills (Item 1), which may not be entirely applicable when assessing pre-clinical subjects such as human anatomy.

### Suggestions for Future Research

This study identified several directions for future research, including evaluating both free and paid anatomy apps. Additionally, increasing the number of raters would ensure the precision of interrater reliability and strengthen evaluation robustness. Furthermore, the authors would like to suggest a modified MARuL tailored to assessing human anatomy education, as there are numerous anatomical apps available on the iOS and Play Store. Finally, it could be beneficial to test the three top-rated apps among undergraduate students to assess their effectiveness in student learning.

## Conclusion

This study contributes to the growing mobile learning literature through the comprehensive analysis of 20 human anatomy mobile applications. Among the evaluated apps, three demonstrated high quality and met the criteria as *probably valuable* by the MARuL scale. Notably, Usability (Part D) emerged as the highest-rated domain across the overall sections. At present, the study demonstrates that the MARuL scale is a reliable evaluation tool for human anatomy apps, particularly in addressing pedagogical requirements of anatomy education. Future studies should assess the top-performing apps for their effectiveness in undergraduate anatomy education.
